# Development and Validation of a Nomogram for Predicting Sepsis Risk in Patients with Non-Ventilator Hospital-Acquired Pneumonia

**DOI:** 10.3390/biomedicines14050987

**Published:** 2026-04-25

**Authors:** Han Zhou, Zhenchao Wu, Beibei Liu, Yipeng Du, Rui Wu, Ning Shen

**Affiliations:** Department of Respiratory and Critical Care Medicine, Peking University Third Hospital, Beijing 100191, China

**Keywords:** non-ventilator hospital-acquired pneumonia, sepsis, risk prediction, nomogram, temporal validation

## Abstract

**Objective**: To identify risk factors for progression to sepsis in patients with non-ventilator hospital-acquired pneumonia (NV-HAP) and to develop a practical nomogram for individualized risk assessment in this population. **Methods**: We retrospectively screened 408 hospitalized patients with hospital-acquired pneumonia at Peking University Third Hospital between January 2017 and December 2021. After excluding patients with an unclear diagnosis date or missing critical variables required for SOFA score calculation, 368 eligible patients with NV-HAP were included and randomly divided into a training cohort (*n* = 260) and an internal validation cohort (*n* = 108). An independent temporal validation cohort of 68 patients admitted between January 2022 and December 2022 at the same center was further used for temporal validation. Univariable and multivariable logistic regression analyses with backward stepwise selection were performed in the training cohort to identify predictors associated with progression to sepsis. A nomogram was then constructed based on the final model and evaluated by discrimination, calibration, and decision curve analysis. **Results**: A total of 368 patients were included in the model development dataset. The final multivariable model retained six predictors: male sex (OR = 2.393, 95% CI: 1.333–4.296), diabetes (OR = 2.205, 95% CI: 1.126–4.319), coagulation dysfunction (OR = 3.327, 95% CI: 1.726–6.413), PaO_2_/FiO_2_ (OR = 0.955 per 10-unit increase, 95% CI: 0.912–1.001), platelet count (OR = 0.900 per 10 × 10^9^/L increase, 95% CI: 0.853–0.949), and bilirubin (OR = 1.176 per 1 μmol/L increase, 95% CI: 1.100–1.258). The nomogram showed acceptable performance, with an apparent C-index of 0.809 and a bootstrap-corrected C-index of 0.792 in the training cohort. The C-index was 0.750 (95% CI: 0.658–0.841) in the internal validation cohort and 0.754 (95% CI: 0.639–0.870) in the temporal validation cohort. Calibration analysis showed acceptable agreement between predicted and observed probabilities, and decision curve analysis indicated a positive net clinical benefit across clinically relevant threshold probabilities. **Conclusions**: In patients with NV-HAP, male sex, diabetes, coagulation dysfunction, lower PaO_2_/FiO_2_, lower platelet count, and higher bilirubin were associated with progression to sepsis. The developed nomogram showed acceptable discrimination, calibration, and clinical utility, and may serve as a practical tool for early individualized risk stratification in patients with NV-HAP.

## 1. Introduction

Sepsis, defined as life-threatening organ dysfunction caused by a dysregulated host response to infection, remains a major global health challenge associated with substantial morbidity and mortality [1]. Despite advances in early recognition and management over the past decade, the overall burden of sepsis remains high because of its complex pathophysiology, nonspecific early manifestations, and rapid clinical deterioration [2,3]. According to the source of infection, sepsis can be broadly categorized as community-acquired or hospital-acquired sepsis [4]. Compared with community-acquired sepsis, hospital-acquired sepsis is generally associated with longer hospital stays, higher mortality, and worse clinical outcomes [4,5].

Pneumonia is one of the most common sources of sepsis [6]. Hospital-acquired pneumonia (HAP), defined as pneumonia occurring 48 h or more after hospital admission and not incubating at the time of admission, is among the most frequent healthcare-associated infections [7]. Clinically, HAP is commonly classified into ventilator-associated pneumonia (VAP) and non-ventilator hospital-acquired pneumonia (NV-HAP) according to the presence or absence of invasive mechanical ventilation [8]. While VAP has been extensively investigated, NV-HAP has received comparatively less attention despite its substantial clinical burden [9,10]. Emerging evidence suggests that NV-HAP is associated with a substantial risk of sepsis that may be underrecognized in routine clinical practice [9,11]. These findings highlight the need to identify predictors of sepsis progression in patients with NV-HAP in order to facilitate earlier recognition and timely management of high-risk individuals.

Several scoring systems, including SIRS, SOFA, qSOFA, and NEWS, are widely used to assess illness severity and prognosis. However, these tools were not specifically developed to estimate the risk of progression to sepsis in patients with NV-HAP [12,13]. In this context, nomograms may provide a practical approach for individualized risk prediction. By integrating multiple clinical variables into an intuitive visual model, nomograms can support bedside risk estimation and improve the clinical applicability of statistical prediction models [14]. Such an approach may be particularly useful in life-threatening infections such as sepsis, in which early identification of high-risk patients is essential for closer monitoring and timely clinical decision-making [15].

Accordingly, this study aimed to identify clinical predictors associated with progression to sepsis in patients with NV-HAP, to develop a disease-specific nomogram for individualized risk assessment, and to evaluate its performance in both internal and independent temporal validation cohorts.

## 2. Methods

### 2.1. Study Population

This retrospective study included 408 patients diagnosed with hospital-acquired pneumonia (HAP) in Peking University Third Hospital between January 2017 and December 2021. After screening the inclusion and exclusion criteria, 368 patients with non-ventilator hospital-acquired pneumonia (NV-HAP) were ultimately included and randomly allocated to a training cohort (*n* = 260) and a validation cohort (*n* = 108). An independent external validation cohort of patients (*n* = 68) was subsequently collected from the same institution between January and December 2022.

Inclusion criteria: (a) Diagnosis consistent with the international guidelines for the management of hospital-acquired pneumonia and ventilator-associated pneumonia [7,8]. (b) Absence of invasive mechanical ventilation before pneumonia onset.

Exclusion criteria: (a) Lower respiratory tract infection already present at admission and subsequently aggravated after hospitalization. (b) New pulmonary infiltrates that could not be differentiated from progression of pre-existing conditions. (c) Incomplete clinical data, insufficient for calculating SOFA scores.

### 2.2. Outcome Definition

The primary outcome was progression to sepsis after the diagnosis of NV-HAP. Sepsis was defined according to the Sepsis-3 criteria as life-threatening organ dysfunction caused by a dysregulated host response to infection. In this study, sepsis was operationalized as an acute increase in the Sequential Organ Failure Assessment (SOFA) score of at least 2 points attributable to infection.

### 2.3. Data Collection

Demographic, clinical, comorbidity, and laboratory data were extracted from the electronic medical record system. Predictor variables were collected during the 48 h immediately before the diagnosis of NV-HAP. Candidate predictors were selected according to clinical relevance and data availability.

The variables evaluated in the derivation dataset included male sex, age, body mass index, hypertension, heart failure, diabetes, respiratory disease, chronic kidney disease, immunosuppression, coagulation dysfunction, hepatic dysfunction, renal dysfunction, hypoproteinemia, Glasgow coma scale, SpO_2_, PaO_2_/FiO_2_, platelet count, and bilirubin. Acute myocardial infarction was initially considered but was excluded from model development because its definition was not uniform across datasets and could introduce misclassification bias.

After the exclusion process, no missing values were present in the variables used for model development in the derivation dataset.

### 2.4. Model Development and Assessment

Univariable logistic regression analyses were first performed in the training cohort to identify variables associated with progression to sepsis. Variables with a *p* value < 0.10 in univariable analyses and considered clinically relevant were entered into a multivariable logistic regression model. Backward stepwise selection was then applied to derive the final model. The final multivariable model retained six predictors: male sex, diabetes, coagulation dysfunction, PaO_2_/FiO_2_, platelet count, and bilirubin. A nomogram was subsequently constructed from the regression coefficients of the final model to provide individualized risk estimation for progression to sepsis in patients with NV-HAP.

The predictive performance of the nomogram was evaluated in the training cohort, the internal validation cohort, and the independent temporal validation cohort. Model discrimination was assessed using the concordance index (C-index), which is equivalent to the area under the receiver operating characteristic curve (AUC) for binary outcomes. In the training cohort, apparent model performance was first assessed. Optimism was then estimated by bootstrap resampling with 1000 iterations to obtain the optimism-corrected C-index.

Calibration was evaluated using calibration plots in the training, internal validation, and temporal validation cohorts. Clinical utility was assessed using decision curve analysis (DCA). The net benefit of the nomogram was compared with the treat-all and treat-none strategies across clinically relevant threshold probabilities. In the temporal validation cohort, the Brier score and the Hosmer–Lemeshow goodness-of-fit test were additionally calculated to further assess model calibration.

### 2.5. Statistical Analysis and Visualization

Categorical variables are presented as *n* (%), and continuous variables are presented as mean ± standard deviation (SD). Comparisons between the sepsis and non-sepsis groups were performed using the chi-square test or Fisher’s exact test for categorical variables and Student’s *t*-test for continuous variables, as appropriate. Odds ratios (ORs) with 95% confidence intervals (CIs) were reported for logistic regression analyses. All tests were two-sided, and *p* < 0.05 was considered statistically significant.

All analyses were performed using R software (version 4.5.2, R Foundation for Statistical Computing, Vienna, Austria). The nomogram was generated using the regplot package. ROC analysis was performed using pROC. DCA was performed using rmda. Figures were generated using ggplot2 and assembled into multi-panel layouts using patchwork.

## 3. Results

### 3.1. Clinical Data

**Figure 1:**
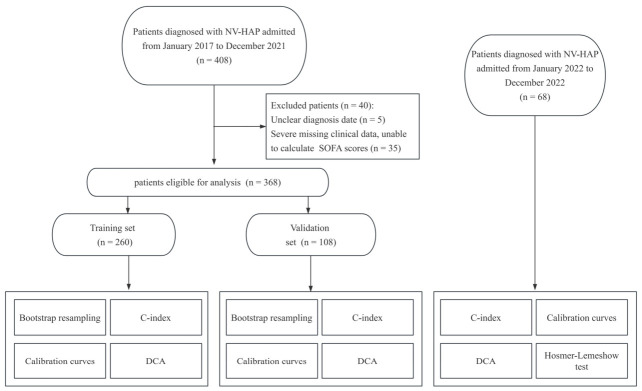
Flowchart of patient enrollment.

Between January 2017 and December 2021, 408 patients with hospital-acquired pneumonia (HAP) were screened. After excluding patients with an unclear diagnosis date (*n* = 5) or missing critical variables required for SOFA score calculation (*n* = 35), 368 eligible patients with non-ventilator hospital-acquired pneumonia (NV-HAP) were included in the final analysis. These patients were randomly divided into a training cohort (*n* = 260) and an internal validation cohort (*n* = 108) at an approximate ratio of 7:3. In addition, an independent temporal validation cohort of 68 patients with NV-HAP admitted between January 2022 and December 2022 at the same center and using the same eligibility criteria was included for temporal validation. The detailed patient enrollment and study flow are shown in Figure 1, and the baseline characteristics of this temporal validation cohort are detailed in Table S1.

As shown in Table 1, significant between-group differences in the training cohort were mainly observed in male sex, respiratory disease, chronic kidney disease, coagulation dysfunction, PaO_2_/FiO_2_, platelet count, and bilirubin level. In the internal validation cohort, respiratory disease, platelet count, and bilirubin were the most prominent discriminating variables. Overall, the main between-group differences were concentrated in selected comorbidities and organ dysfunction-related variables, particularly oxygenation status, platelet count, and bilirubin level.

### 3.2. Risk Factors for Sepsis in Patients with Non-Ventilator Hospital-Acquired Pneumonia

To identify factors associated with progression to sepsis, univariable logistic regression analyses were first performed in the training cohort (*n* = 260), including 136 patients in the sepsis group and 124 in the non-sepsis group. Variables with a *p* value < 0.10 in univariable analyses and considered clinically relevant were entered into a multivariable logistic regression model, and backward stepwise selection was then applied to derive the final model.

The final multivariable model retained six predictors: male sex, diabetes, coagulation dysfunction, PaO_2_/FiO_2_, platelet count, and bilirubin (Table 2). The detailed definitions and clinical interpretations of these predictive variables are provided in Table S2. Male sex (OR 2.393, 95% CI 1.333–4.296; *p* = 0.003), diabetes (OR 2.205, 95% CI 1.126–4.319; *p* = 0.021), coagulation dysfunction (OR 3.327, 95% CI 1.726–6.413; *p* < 0.001), lower platelet count (OR 0.900 per 10 × 10^9^/L increase, 95% CI 0.853–0.949; *p* < 0.001), and higher bilirubin level (OR 1.176 per 1 μmol/L increase, 95% CI 1.100–1.258; *p* < 0.001) were independently associated with progression to sepsis. A lower PaO_2_/FiO_2_ ratio showed a directionally consistent but borderline association in the multivariable model (OR 0.955 per 10-unit increase, 95% CI 0.912–1.001; *p* = 0.057). Although respiratory disease and chronic kidney disease were associated with sepsis status in baseline comparisons, they were not retained in the final multivariable model after adjustment for coexisting predictors and backward stepwise selection.

### 3.3. Development and Validation of a Nomogram for Predicting Progression to Sepsis in Patients with Non-Ventilator Hospital-Acquired Pneumonia

**Figure 2:**
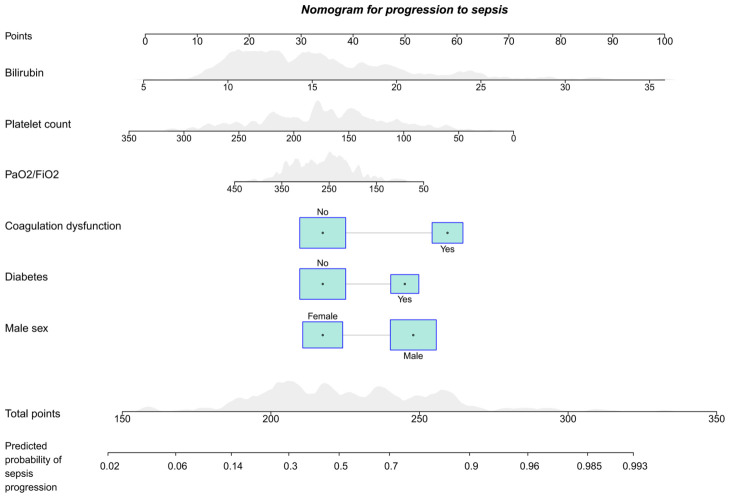
Sepsis Risk Nomogram.

Based on the final multivariable logistic regression model derived from the training cohort, we constructed a nomogram incorporating six predictors: male sex, diabetes, coagulation dysfunction, PaO_2_/FiO_2_, platelet count, and bilirubin (Figure 2). Each variable was assigned a weighted score, and the total score was used to estimate the individual probability of progression to sepsis.

**Figure 3:**
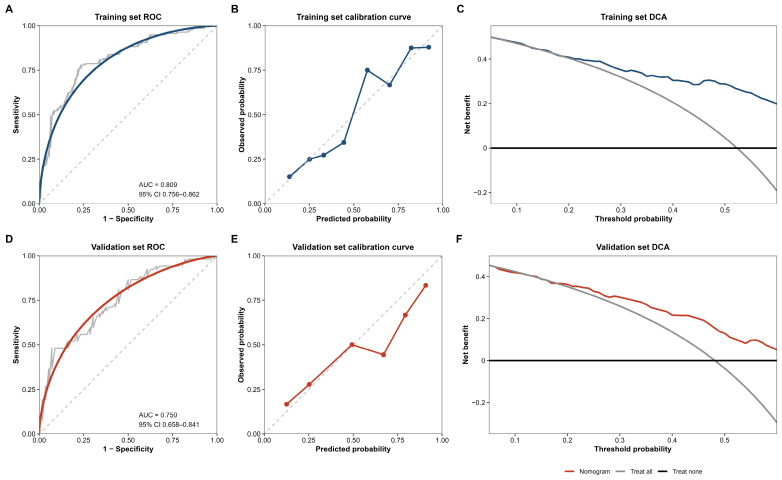
Performance of the nomogram in the training and internal validation cohorts. (**A**) Training ROC curve. (**B**) Training calibration curve. (**C**) Training decision curve analysis. (**D**) Internal validation ROC curve. (**E**) Internal validation calibration curve. (**F**) Internal validation decision curve analysis. In the ROC and calibration curves, the dashed grey line represents the reference line for random chance (AUC = 0.5) and ideal perfect prediction, respectively. In the calibration curves, the dark blue and red dots represent the observed versus predicted probabilities for each risk interval in the training and validation sets, respectively. In the DCA curves, the dark blue and red solid lines represent the net benefit of the nomogram, while the solid grey and black lines represent the treat-all and treat-none strategies, respectively.

The apparent C-index of the nomogram in the training cohort was 0.809. After bootstrap resampling (1000 iterations), the optimism-corrected C-index was 0.792, indicating acceptable internal stability of the model. In the internal validation cohort, the nomogram showed moderate discrimination, with a C-index of 0.750 (95% CI 0.658–0.841). Calibration plots showed acceptable agreement between predicted and observed probabilities in both the training and internal validation cohorts (Figure 3). Decision curve analysis further suggested that the nomogram provided a positive net benefit over the treat-all and treat-none strategies across a clinically relevant range of threshold probabilities (Figure 3).

The predictive performance of the nomogram was further evaluated in an independent temporal validation cohort from the same center (*n* = 68). In this cohort, the nomogram achieved a C-index of 0.754 (95% CI 0.639–0.870), with a Brier score of 0.203. The calibration curve showed acceptable agreement between predicted and observed probabilities, and decision curve analysis demonstrated a consistent net benefit over clinically relevant threshold probabilities. In addition, the Hosmer–Lemeshow goodness-of-fit test showed no evidence of poor fit (χ^2^ = 3.95, df = 3, *p* = 0.535), supporting acceptable calibration of the nomogram in the temporal validation cohort (Supplementary Figure S1).

## 4. Discussion

In this retrospective single-center study, we identified six predictors associated with progression to sepsis in patients with NV-HAP, namely male sex, diabetes, coagulation dysfunction, PaO_2_/FiO_2_, platelet count, and bilirubin level. Based on these variables, we developed a disease-specific nomogram for individualized risk assessment and further evaluated its performance in both an internal validation cohort and an independent temporal validation cohort from the same center. The model showed acceptable discrimination, calibration, and clinical utility, suggesting that it may support early risk stratification in this clinical setting.

Among baseline clinical characteristics, male sex was independently associated with a higher risk of progression to sepsis. Previous studies have suggested that sex-related differences in immune regulation and hormonal responses may contribute to variations in susceptibility to severe infection, with male patients potentially being more vulnerable to bacterial infection and sepsis [16,17]. Diabetes was also retained in the final model. This association is biologically plausible, as diabetes is linked to impaired innate and adaptive immune responses, altered inflammatory regulation, and increased susceptibility to infection, all of which may predispose patients with NV-HAP to systemic deterioration once infection occurs [18].

Coagulation dysfunction was another important predictor in our model. This finding is consistent with the recognized role of sepsis-associated coagulopathy in microvascular thrombosis, impaired tissue perfusion, and subsequent organ dysfunction [19,20]. In the context of NV-HAP, early coagulation abnormalities may reflect both infection severity and an evolving dysregulated host response, thereby providing clinically meaningful information beyond baseline comorbidity alone.

The laboratory predictors identified in the final model were also in line with the pathophysiological processes underlying sepsis progression. A lower PaO_2_/FiO_2_ ratio reflects impaired pulmonary gas exchange and more severe respiratory compromise, which is particularly relevant in a pneumonia-based cohort [21]. Although the association of PaO_2_/FiO_2_ in the multivariable model was borderline, its direction was consistent with clinical expectations and with the between-group differences observed at baseline. Lower platelet count was independently associated with progression to sepsis, in agreement with previous evidence linking thrombocytopenia to coagulation abnormalities, inflammatory consumption, and poor outcomes in sepsis [22,23]. Higher bilirubin level was also associated with increased risk, suggesting that early hepatic dysfunction or cholestatic disturbance may already be present in patients at higher risk of systemic deterioration [24]. Taken together, these findings indicate that abnormalities in oxygenation, hematologic status, and bilirubin may serve as early warning signals for sepsis progression in patients with NV-HAP.

An important strength of this study is that it moved beyond identifying isolated correlates and translated the retained predictors into a practical bedside risk assessment tool. Existing scoring systems such as SIRS, SOFA, qSOFA, and NEWS are widely used for illness severity assessment or prognostic evaluation, but they were not specifically developed to estimate the risk of progression to sepsis in patients with NV-HAP. In contrast, our nomogram was tailored to this clinical scenario and incorporated routinely available variables into a simple visual model. Rather than replacing established clinical scoring systems, this nomogram may serve as a complementary tool for early individualized risk stratification in patients with NV-HAP.

Several limitations should be acknowledged. First, this was a retrospective single-center study with a relatively limited sample size, which may restrict the generalizability of the findings. Second, although we included variables that were clinically accessible and practical, some potentially relevant factors, such as dynamic inflammatory markers, microbiological characteristics, and treatment-related variables, were not incorporated into the final model. Third, although the model was further assessed in an independent temporal cohort, both derivation and validation data were obtained from the same center; therefore, true multicenter external validation is still lacking. Fourth, some retained predictors, particularly PaO_2_/FiO_2_, platelet count, and bilirubin, are closely related to early organ dysfunction and should therefore be interpreted as markers supporting early risk stratification rather than long-horizon prediction far removed from clinical deterioration. Finally, some candidate variables, such as acute myocardial infarction, could not be retained because their definition was not fully uniform across datasets, highlighting the importance of standardized variable ascertainment in future studies.

## 5. Conclusions

In conclusion, male sex, diabetes, coagulation dysfunction, lower PaO_2_/FiO_2_, lower platelet count, and higher bilirubin were associated with progression to sepsis in patients with NV-HAP. The resulting nomogram demonstrated acceptable performance in internal and temporal validation and may provide a practical tool for early individualized risk assessment. Further prospective multicenter studies are warranted to confirm its robustness, transportability, and clinical utility.

## Figures and Tables

**Table 1:** Baseline characteristics of patients with and without sepsis in the training and validation cohorts.

Variables	Training Cohort (*N* = 260)			Validation Cohort (*N* = 108)		
	Sepsis (*n* = 136)	Non-Sepsis (*n* = 124)	*p* Value	Sepsis (*n* = 52)	Non-Sepsis (*n* = 56)	*p* Value
Demographic						
Male sex	91 (66.91%)	57 (45.97%)	<0.001	30 (57.69%)	29 (51.79%)	0.538
Age (years)	68.80 ± 9.71	70.31 ± 10.72	0.238	67.94 ± 9.65	69.80 ± 11.34	0.359
BMI (kg/m^2^)	24.90 ± 3.76	24.63 ± 3.54	0.561	24.36 ± 3.67	23.66 ± 3.36	0.308
Major comorbidities						
Hypertension	69 (50.74%)	60 (48.39%)	0.705	28 (53.85%)	28 (50.00%)	0.689
Heart failure	29 (21.32%)	24 (19.35%)	0.694	8 (15.38%)	13 (23.21%)	0.304
Diabetes	44 (32.35%)	27 (21.77%)	0.056	12 (23.08%)	20 (35.71%)	0.151
Respiratory disease	44 (32.35%)	23 (18.55%)	0.011	21 (40.38%)	7 (12.50%)	<0.001
Chronic kidney disease	16 (11.76%)	5 (4.03%)	0.022	5 (9.62%)	2 (3.57%)	0.258
Immunosuppression	13 (9.56%)	8 (6.45%)	0.358	5 (9.62%)	3 (5.36%)	0.478
Clinical conditions						
Coagulation dysfunction	57 (41.91%)	24 (19.35%)	<0.001	21 (40.38%)	18 (32.14%)	0.373
Hepatic dysfunction	12 (8.82%)	7 (5.65%)	0.325	9 (17.31%)	6 (10.71%)	0.322
Renal dysfunction	12 (8.82%)	15 (12.10%)	0.388	6 (11.54%)	4 (7.14%)	0.517
Hypoproteinemia	45 (33.09%)	50 (40.32%)	0.226	22 (42.31%)	22 (39.29%)	0.749
Glasgow coma scale	13.16 ± 2.60	13.26 ± 2.64	0.768	13.23 ± 2.43	13.11 ± 2.40	0.791
Vital signs/laboratory findings						
SpO_2_ (%)	93.86 ± 2.88	93.72 ± 2.73	0.682	94.37 ± 2.54	93.96 ± 2.90	0.446
PaO_2_/FiO_2_	252.32 ± 68.72	275.87 ± 56.28	0.003	259.79 ± 65.85	279.10 ± 67.62	0.136
Platelet count (×10^9^/L)	158.03 ± 55.63	190.91 ± 57.04	<0.001	152.69 ± 58.74	195.34 ± 54.87	<0.001
Bilirubin (μmol/L)	17.17 ± 5.73	13.92 ± 4.16	<0.001	18.12 ± 5.27	13.62 ± 4.72	<0.001

**Table 2:** Univariable and multivariable logistic regression analyses for progression to sepsis in the training cohort.

Variable	Univariable OR (95% CI)	*p* Value	Multivariable OR (95% CI)	*p* Value
Male sex	2.377 (1.438–3.928)	<0.001	2.393 (1.333–4.296)	0.003
Diabetes	1.718 (0.984–3.001)	0.057	2.205 (1.126–4.319)	0.021
Coagulation dysfunction	3.006 (1.716–5.267)	<0.001	3.327 (1.726–6.413)	<0.001
PaO_2_/FiO_2_ (per 10-unit increase)	0.942 (0.905–0.981)	0.004	0.955 (0.912–1.001)	0.057
Platelet count (per 10 × 10^9^/L increase)	0.902 (0.861–0.944)	<0.001	0.900 (0.853–0.949)	<0.001
Bilirubin (per 1 μmol/L increase)	1.145 (1.082–1.212)	<0.001	1.176 (1.100–1.258)	<0.001

## Data Availability

The datasets generated and analyzed during this study are available from the corresponding author upon reasonable request.

## Supplementary Materials

The following supporting information can be downloaded at: https://www.mdpi.com/article/10.3390/biomedicines14050987/s1, Figure S1: Performance of the nomogram model in the temporal validation cohort, including (**A**) Receiver operating characteristic (ROC) curve, (**B**) Calibration curve, and (**C**) Decision curve analysis (DCA); Table S1: Baseline characteristics of patients with and without sepsis in the temporal validation cohort; Table S2: Definition and clinical interpretation of the predictive variables incorporated in the nomogram.
