# Supplementary material for: Development and Validation of a Nomogram for Predicting Sepsis Risk in Patients with Non-Ventilator Hospital-Acquired Pneumonia
Source: Biomedicines. 2026 Apr 25;14(5):987. doi: 10.3390/biomedicines14050987 (PMC13204423; doi:10.3390/biomedicines14050987)
Supplement: Supplementary file 1 [file biomedicines-14-00987-s001.zip › Supplementary Table S1 and S2.pdf]

Table S1. Baseline characteristics of patients with and without sepsis in the temporal validation cohort

**Variables**	**Temporal validation cohort (*N*=68)**		
	**Sepsis (*n*=35)**	**Non-sepsis (*n*=33)**	***p* value**
Demographic			
Male sex	21 (60.00%)	17 (51.52%)	0.646
Age (years)	68.29 ± 9.79	65.30 ± 9.04	0.196
BMI (kg/m^2)	24.67 ± 3.11	25.14 ± 3.30	0.543
Major comorbidities			
Hypertension	22 (62.86%)	16 (48.48%)	0.343
Heart failure	2 (5.71%)	7 (21.21%)	0.127
Diabetes	4 (11.43%)	14 (42.42%)	0.009
Respiratory disease	9 (25.71%)	4 (12.12%)	0.264
Chronic kidney disease	7 (20.00%)	2 (6.06%)	0.181
Immunosuppression	3 (8.57%)	3 (9.09%)	>0.999
Clinical conditions			
Coagulation dysfunction	11 (31.43%)	8 (24.24%)	0.697
Hepatic dysfunction	3 (8.57%)	2 (6.06%)	>0.999
Renal dysfunction	6 (17.14%)	2 (6.06%)	0.298
Hypoproteinemia	12 (34.29%)	17 (51.52%)	0.234
Glasgow coma scale	12.31 ± 3.57	13.00 ± 2.94	0.389
Vital signs / laboratory findings			
SpO_2 (%)	94.00 ± 3.00	94.00 ± 2.00	0.509
PaO_2/FiO_2	231.89 ± 73.94	295.95 ± 58.13	<0.001
Platelet count (×10^9/L)	155.60 ± 59.53	209.79 ± 50.41	<0.001
Bilirubin (μmol/L)	16.61 ± 5.18	13.55 ± 2.74	0.003

Note: Data are presented as *n* (%) for categorical variables and mean ± standard deviation (SD) for continuous variables. Comparisons between the sepsis and non-sepsis groups were performed using the chi-square test or Fisher's exact test for categorical variables and the Student's t-test for continuous variables, as appropriate. BMI, body mass index; SpO_2, peripheral oxygen saturation; PaO_2/FiO_2, arterial partial pressure of oxygen to fraction of inspired oxygen ratio. Coagulation dysfunction, hepatic dysfunction, and renal dysfunction were defined according to the prespecified clinical criteria recorded in the study database.

Table S2. Definition and clinical interpretation of the predictive variables incorporated in the nomogram

Variable	Value Type / Unit	Definition & Measurement	Thresholds & Clinical Interpretation (SOFA)
Male sex	Binary (1/0)	Male = 1, Female = 0.<br>Documented biological sex at birth.	Associated with sex-related differences in immune regulation and susceptibility to severe infection.
Diabetes	Binary (1/0)	Documented pre-existing diagnosis of diabetes mellitus or current use of anti-diabetic medications.	Reflects impaired innate/adaptive immune responses and altered inflammatory regulation.
Coagulation dysfunction	Binary (1/0)	Activated Partial Thromboplastin Time (APTT) > 40s, OR D-dimer > 2.0 μg/mL (FEU).	Normal: APTT 25–35s; D-dimer <0.5 μg/mL.<br><br>Interpretation: Indicative of early sepsis-associated coagulopathy and systemic activation.
PaO_2/FiO_2<br>(per 10-unit increase)	Continuous (mmHg)	Arterial oxygen partial pressure (PaO_2) divided by the fraction of inspired oxygen (FiO_2).	SOFA Respiratory Subscore: ≥400: Normal (0); 301–399: Mild (1); 201–300: Moderate (2); 101–200: Severe (3); ≤100: Very severe (4)
Platelet count<br>(per 10×10^9/L increase)	Continuous ($\times10^9$/L)	Measured from complete blood count during the 48 h immediately before NV-HAP diagnosis.	SOFA Coagulation Subscore: ≥ 150: Normal (0); 100–149: Mild decrease (1); 50–99: Moderate (2); 20–49: Severe (3); < 20: Very severe (4)
Bilirubin<br>(per 1 μmol/L increase)	Continuous (μmol/L)	Total serum bilirubin level measured during the 48 h immediately before NV-HAP diagnosis.	SOFA Hepatic Subscore: < 20: Normal (0); 20–32: Mild elevation (1); 33–101: Moderate (2); 102–204: Severe (3); > 204: Very severe (4)

Note: All laboratory and clinical variables were collected during the 48 hours immediately before the diagnosis of NV-HAP. If multiple measurements were available within this window, the value closest to the time of NV-HAP diagnosis was selected for analysis. Definitions, units, and threshold values were determined in reference to standard clinical practice and the Sepsis-3 consensus definition. These six variables were independently associated with progression to sepsis and were retained in the final multivariable model.
