# Supplementary figures and images for: Development and Validation of a Nomogram for Predicting Sepsis Risk in Patients with Non-Ventilator Hospital-Acquired Pneumonia
Source: Biomedicines. 2026 Apr 25;14(5):987. doi: 10.3390/biomedicines14050987 (PMC13204423; doi:10.3390/biomedicines14050987)

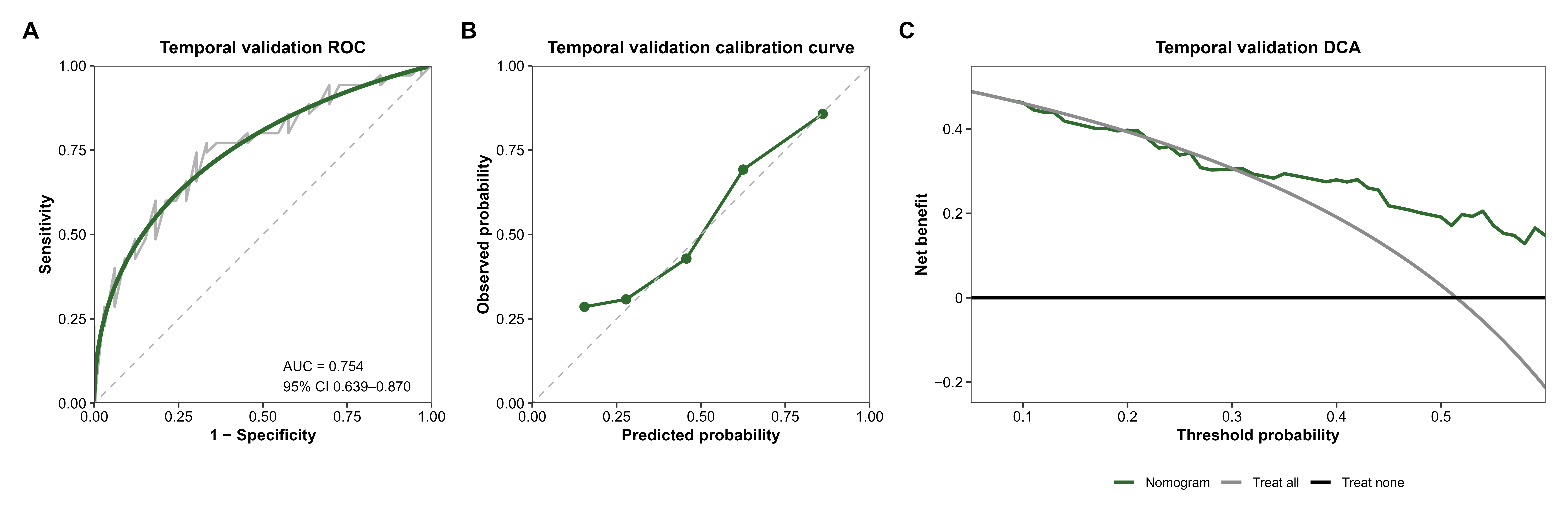

Supplement: Supplementary file 1 [file biomedicines-14-00987-s001.zip › Supplementary Figure S1.png]
